# An alternative, easy and reproducible method of stabilization and ligature-induced periodontitis in mouse

**DOI:** 10.1016/j.mex.2019.09.004

**Published:** 2019-09-17

**Authors:** Suiane S.C. Pereira, Geisoellen F. Araujo, Lucas N. de Queiroz, Priscilla R. Câmara, Vinícius D.B. Pascoal, Rebeca S. Azevedo, Bruno K. Robbs

**Affiliations:** a Postgraduate Program in Dentistry, Health Institute of Nova Friburgo, Fluminense Federal University, Nova Friburgo, Brazil; b Postgraduate Program in Applied Science for Health Products, Faculty of Pharmacy, Fluminense Federal University, Niteroi, Brazil; c Postgraduate Program in Dentistry, School of Dentistry, Fluminense Federal University, Niterói, RJ, Brazil; d Basic Science Department, Health Institute of Nova Friburgo, Fluminense Federal University, Nova Friburgo, Brazil; e Oral Pathology, Dentistry Faculty, Health Institute of Nova Friburgo, Fluminense Federal University, Nova Friburgo, Brazil

**Keywords:** Ligature-induced periodontitis, Periodontal disease, Ligature, Animal model, Method, Mice, Immobilization, Bone loss

## Abstract

Periodontal disease is one of the most common causes of tooth loss in the world. Ligature-induced is the most used method to study periodontitis. Here, we describe a alternative, easy and accessible experimental technique of ligation in mice. Twenty C57BL/6 female mice were divided in two groups, control and ligation. Ligature group (n = 10) was immobilized in a well described stabilization board and ligature was performed at the first molar using a new procedure here described in detail. Eight weeks later animals were euthanized, and periodontitis hallmarks were evaluated. Ligatures remained attached to the teeth in all animal during the hole experiment. The procedure induced a temporary loss of weight but no causalities or tooth loss. The animals affected by ligation in their molar teeth presented all periodontitis hallmarks, including alveolar bone loss, gingival retraction and inflammatory infiltrate in the studied region both macro and microscopically. The alternative method is low cost, easily reproducible, and induces all periodontitis hallmarks that are sustained until 8 weeks after placement.

• Ligature-induced periodontitis in mouse is a powerful tool of research.
• Methods describing the procedure in literature are difficult to reproduce.
• A alternative stabilization and ligation procedure in mice is completely described here.

## Graphical abstract

**Figure:**
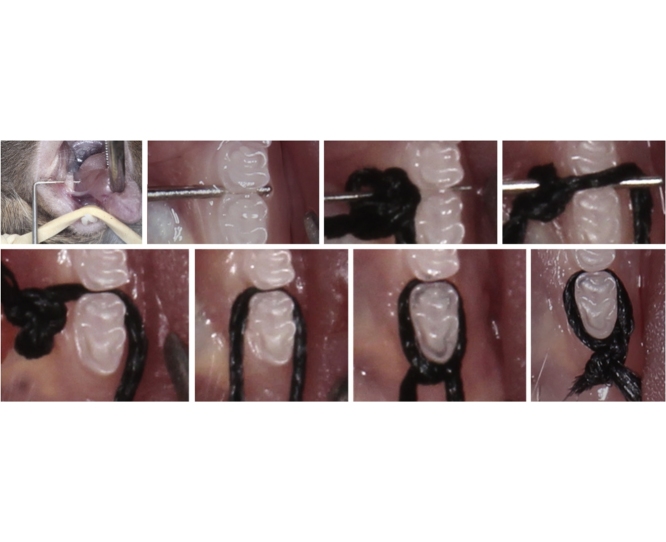

**Specification Table**

**Table:** 

Subject Area:	Medicine and Dentistry
More specific subject area:	Periodontics research
Method name:	Ligature-induced periodontitis
Name and reference of original method:	Our method is an enhanced version of the Ligature-induced periodontitis protocol described in: “T. Abe and G.Hajishengallis, Optimization of the ligature-induced periodontitis model in mice. J. Immunol. Methods3941-220134954”. Our method describes in detail the construction and application of a stabilization apparatus for mice oral surgery and an easy, reproducible method to induce periodontitis using easy to acquire material for ligation procedure.
Resource availability:	NA

## Method details

There are several models of induction of periodontitis in the literature, such as bacterial infection model with *Aggregatibacter actinomycetemcomitans*, oral gavage, lipopolysaccharide injection, calvarial model and ligature in molar teeth performed in lower first molars as thoroughly revised and exemplified in Graves et al. 2011 and others [[1], [2], [3]]. The method of ligature in mice has several advantages over other techniques of periodontal disease induction such as that requires less specialized equipment and technique to grow anaerobic bacteria and lead to rapid disease induction, predictable bone loss at a specific site of study and the capacity to study periodontal tissue and alveolar bone regeneration because the model is established within the periodontal apparatus that can be removed [[4], [5], [6], [7], [8], [9]]. Since ligature induces acute bone resorption there is the possibility of regeneration of the alveolar bone in animals that loses spontaneously the ligature during the experimental time. To avoid this artifact the correct tight fixation of ligature around the tooth is very important.

However, our group and others to which we had contact in conferences and technical collaborations had difficulty to reproduce this technique in their labs in mice, to obtain the appropriate material, to immobilize the mouse for the procedure, to produce ligature that remained in place for a long period and to train personnel able to replicate the method. As this is a subjective parameter depending in the materials used, the operator dexterity and experience in handling the mouse model of oral affections this difficulty might justify the preference to use larger animal model such as rats [10]. Ligation technique in mice is not well explored in the literature with detailed protocols, which makes it difficult to perform in independent laboratories, so it is extremely important in the scientific milieu to delimit well described and easily performed mechanisms for the exploitation of this technique and its knowledge [6,11].

### Animals

The study of periodontal disease through the ligation method was performed on females of twelve weeks old C57BL/6 mice weighing approximately 25 g. The project was approved by the ethical council of animal use of the Federal University of Fluminense under registration number 978. All experiments were carried out in accordance with the Brazilian guidelines and regulations for animal use. For the experiment, 20 animals were divided into two groups. The control group (n = 10) had no ligatures on the teeth and the studied group with ligation (n = 10) received ligature in the lower first molars bilaterally. Ligature were maintained for 8 weeks after placement. This time is relevant for the study to prove the ability to cause periodontal disease or not in the animals studied, considering other studies that may stimulate periodontitis with more than eight weeks.

### Stabilization of animals for ligation

**Fig. 1:**
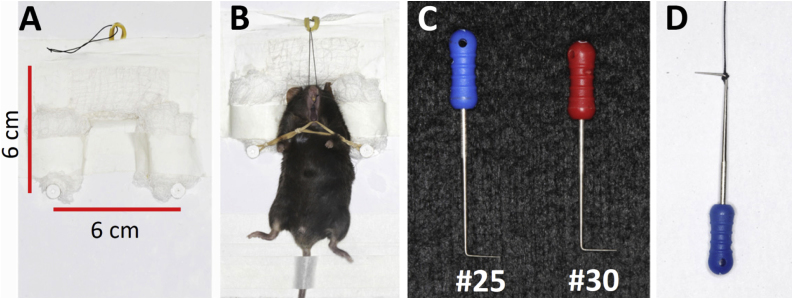
Material used for mouse stabilization and ligation placement. (A) For stabilization of the animals a white plate of wood board with the following dimensions: 14.0 x 18.0 cm (width x height) was used. Gauze were affixed to the upper portion and side portions of the plate with white adhesive tapes to stabilize the animal. An elastic was inserted at the top along with 5-0 silk wire to fix the animal's jaw. Parallel screws in the central portion of the plaque as shown by the arrows for elastic placement to keep the animal's jaw open. Measurements are 6 cm from the bolt to the upper elastic, 6 cm between each bolt and 9 cm between the bolt and the base of the plate. (B) Stabilization of the animal for application of the ligation technique. Fixation of silk suture 5-0 in the upper portion to give opening of the animal's jaw. Elastic in the horizontal portion between the screws, allowing the elastic to be stretched to the lower incisors to give better opening and facilitate access of the animal's mouth. White adhesive tape on the animal's tail to avoid large movements during the procedure. Gazes positioned around the body of the animal to allow flexibility and absence of exacerbated movements during the application of the ligature. (C) Spacers number 30 and 25 to perform the technique with Soft fold at the tip of the spacer at a 90-degree angle to give greater skill to the technique and adhesion to the suture. (D) Suture wire attached to spacer number 25.

For stabilization of the animals a white wood board with the following dimensions: 14.0 x 18.0 cm (width x height) was used. In the middle upper portion of the plate an orifice was made where an elastic was pierced in which a 5-0 suture was connected. This suture had the function of allowing the mouse maxillary opening through its upper incisor teeth. In the middle of the plate were placed two Phillips number 8 screws with a distance of 6.0 cm from each other, with the function of supporting a natural rubber elastic. This elastic was used to open the animal's mandible, supported on the lower incisors (Fig. 1A). For the fixation of the mouse head and lateral portion, gauze stabilized with white adhesive tape was used to soften and not cause damage to the animal and increase stability allowing less movement during the technique (Fig. 1B).

### Materials used for ligature placement in tooth

Finger Endo Spreaders number 25 and 30 (Premier Dental, PA; USA); Castroviejo Needle Holder Straight Tip Serrated Jaws with TC Inserts (Model SKU: 36-1360TC; Surgical 123, NJ; USA); Micro Iris Scissors Straight Blades Sharp Points (Model SKU: 48-2840S; Surgical 123, NJ; USA); Silk 5-0 dental suture thread (Model SUT107421; Roboz Surgical, MD; USA).

### Ligature placement

**Fig. 2:**
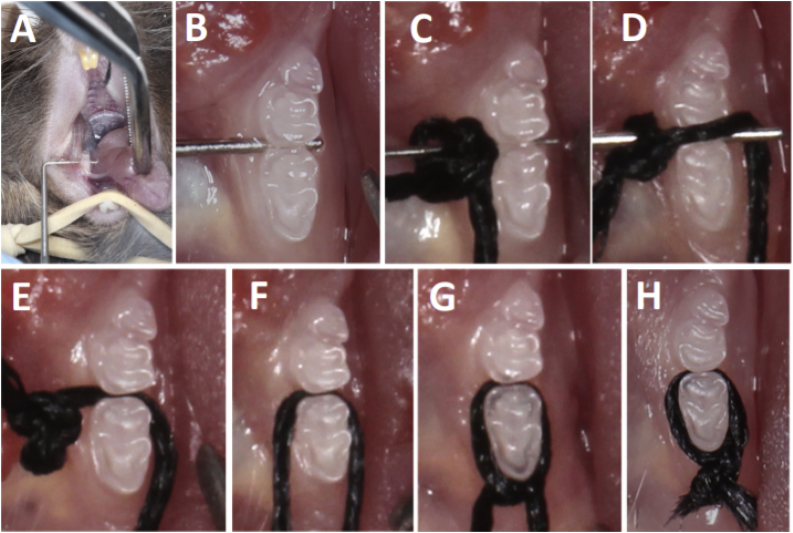
Technical ligation procedures. (A) Opening of the region between the first and second molar teeth through the number 30 finger spreader. (B) spacer number 30 between molar teeth for interdental opening. (C) Suture wire attached to the number 25 spacer folded at its tip ready to be passed between the molars of the animal. (D) The suture is drawn to the other side of the interdental space being pressed by the tip of the spacer after the knot is slightly attached to the cervical region of the tooth. (E) After the wire is firm between the teeth, the spacer is removed. (F) The wire is parallel to each other with the tips facing the anterior side of the teeth. (G) With the use of the hands the first knot is performed in order to test that the wire is firmly in the cervical region of the tooth. (H) Triple-knot completed and use of scissors to cut the silk strand 5-0. Bandages were maintained for 8 weeks until euthanasia. After euthanasia, the mandibles were removed and cut in half, dividing into the direct hemi and the left jaw of both groups, as shown in Fig. 3.

**Fig. 3:**
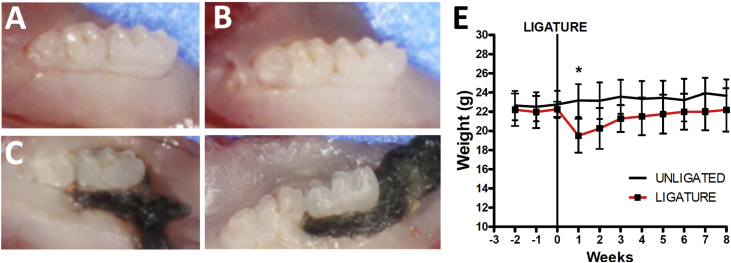
Jaws removed after euthanasia of the two groups and cut in half for later study of the sample for delimitation of the bone and histological loss of the tissue. (A and B) Jaw of the control group. (C and D) Jaw of the ligature group. (E) Comparing the weight among the groups studied, showing weight loss of the group that obtained ligation after one week of laying of the thread. This weight returned to normal and remained stable until the last week. Data are means ± S.D. * represents p < 0.05 compared to non-ligated baseline control.

The animals were sedated with ketamine / xylazine (100/10 mg / kg) intraperitoneally and placed on the stabilizer plate and lightly fixed with white adhesive tape in the syrup and abdomen. Before the beginning of the technique, it was necessary to make a bend in the finger spreaders. With forceps a small bend was made at 3 mm from the spreaders tip in a 90 degrees angle (Fig. 1C), were the silk suture tread was tied with a knot at spreader 25 (Fig. 1D). The folded tip of spacer number 30 was positioned between the first and second molars leading to a slight gap between these teeth (Fig. 2A–B). The spreader 25 with the 5-0 suture tread was crossed between the molars (Fig. 2C) and was lightly pressed, so that, its tip was inserted carefully between the interdental (first and second molar) space and the knot located in the spacer 25 is pierced to the opposite side in order to adhere to the cervical region of the molar tooth (Fig. 2D). Then the Castroviejo forceps was used to pull the tip of the thread that remained on the pierced side followed by removal of the spacer from the animal's mouth (Fig. 2E). With the Castroviejo tweezers both ends were drawn leaving them parallel when they were cut with the Micro iris scissors allowing the knot to be given (Fig. 2F). The sutures were hand held and the manipulator easily performed the first knot on the mesial of the first molar tooth (Fig. 2G). After checking whether the first knot was well attached to the cervical region of the tooth, the operator performed two more knots in order to obtain a triple-knot at the end of the procedure (2 H). The Castroviejo clamp was used to help stabilize the knots. Animals were weighed weekly for 8 weeks after ligature placement (Fig. 3A–D). Ligature were placed in both sides of the jaw in each animal. The ligatures remained in place in all mice throughout the experimental period (Fig. 3E).

### Determination of bone loss induced by ligation

**Fig. 4:**
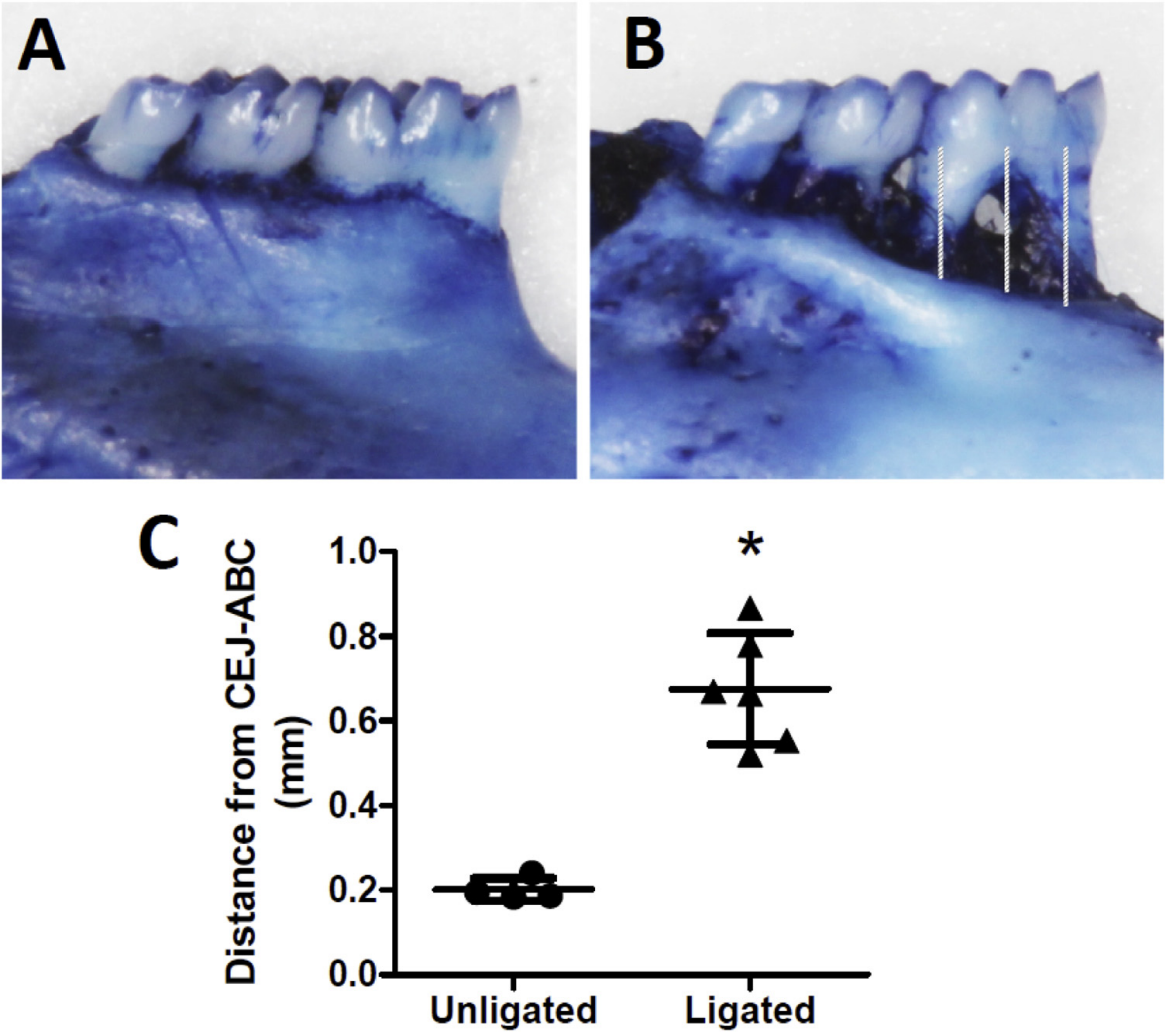
Induction of periodontitis by ligation leads to bone loss of the first molar, observed by staining with methylene blue. (A) Representation of the jaws of the control group. (B) Representation of the jaws of the ligature group. (C) Graphic representation of the bone loss analysis of each group, demonstrated by the cement-enamel junction distance (CEJ-ABC). Total alveolar bone loss was considered as the mean between the mesial and distal roots measurements in relation to the height of the bone crest. The graph represents the individual results for each animal, the mean ± standard deviation. * represents p < 0.001.

After 8 weeks, animals were euthanized for macroscopic bone loss analysis. The jaws were removed and the left hemi jaws were used for the macroscopic analysis of bone loss (Fig. 4A–C). For complete removal of soft tissues, the hemi jaws were immersed in 30% H_2_O_2_ for 16 h after removal of excess tissue with scalpel. The hemi jaws were dried at room temperature, stained for 30 min with 3% methylene blue. Extraction of the excess methylene blue was done with gauze and distilled water with subsequent drying of this material.

The hemi jaws were photographed with Canon Ultrasonic DS126151 camera (10.1 megapixels), using a millimeter ruler as scale. ImageJ Software (NIH, USA) was used to draws and measure three series of lines on the first molar between cementoenamel junction (CEJ) to the alveolar bone crest (ABC). Three points were measured, one at the middle of each root and one in the space between them and the means were calculated (Fig. 4A–C). A statistical analysis was used by the Prisma GRAPHPAD 5.0 ​​program (Intuitive Software for Science, San Diego, CA).

### Histopathology

The right hemi jaw was decalcified in saline (PBS) containing 0.5 EDTA and 3% formaldehyde for at least 4 weeks. These samples were immediately sectioned in the mesiodistal direction, placed in previously identified histological cassettes and then washed in running water for at least 30 min. Afterwards, the samples were processed using an automatic tissue processor (PT09, Lupetec, Sao Carlos, Brazil), and included in liquid paraffin to obtain the histological blocks. Histological 5-μm sections were obtained by the use of a rotating microtome (RM2125 RTS, Leica Biosystemns) and stained with hematoxylin and eosin for viewing under a light microscope.

### Method validation

After animal stabilization (Fig. 1A–D) and ligature placement (Fig. 2A–H), both described in detail in method section, ligatures were left for eight weeks. The ligatures remained in place in all mice in both sides throughout the experimental period (Fig. 3A–D). One week after ligature placement, the animal group with ligature displayed weight loss in relation to the control group, but rapidly returned to the control group weight (Fig. 3E).

After the euthanasia, it was readily noticed a difference between the studied groups, since the control group showed no loss of gingival insertion or alteration in the structural morphology of the studied area on both jaw sides (Fig. 3A–D). Furthermore, food impaction was observed in the group receiving ligation, causing food accumulation in the gingival sulcus and increased gingival recession on both sides (Fig. 3C–D).

To assess bone loss, the CEJ-ABC distances were measured at 3 predetermined sites in the palatal surface (Fig. 4A). While there was no apparent bone loss in control animals, the ligation animals induced a high degree of bone loss when compared with the control one (Fig. 4A–C). There was a large apical displacement of the gingival margin in relation to the CEJ with consequent exposure of the root surface on the group that received the ligature in its first molars (Fig. 4B). Total alveolar bone loss was considered as the mean between the mesial and distal roots measurements in relation to the height of the bone crest (Fig. 4C). These data corroborate the hypothesis that the ligation technique here described induces the appearance of periodontal disease, characterized by structural alteration of the normal dental pattern and the loss of the dental support apparatus, such as the periodontal ligament, as well as, the loss of the insertion of the fibers that keep the tooth adhered to the bone in the area involved by the ligature.

**Fig. 5:**
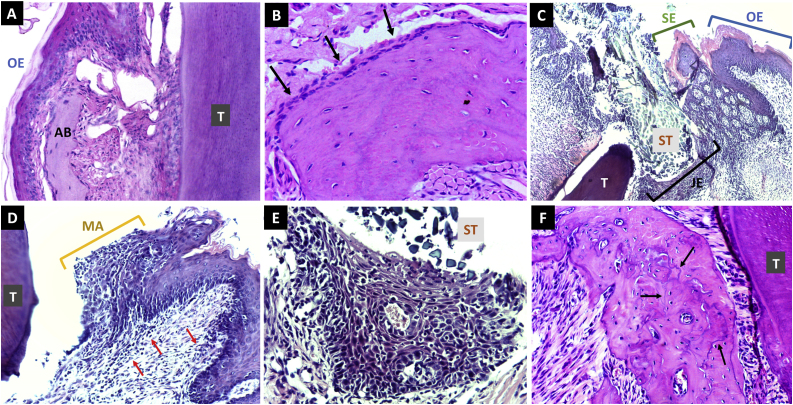
Histopathological microscopic analysis. Representative images of histopathological analyzes stained with hematoxylin and eosin. In (A–B) control group; in (C–F) ligature group. A) In this microscopic image of the control group it’s possible to observe oral epithelium (OE) alveolar bone (AB) and tooth root (T). Note that there is no presence of inflammation (objective x20); B) In this microscopic picture it’s possible to observe the rime of osteoblasts in the periphery from the alveolar bone (objective x40); C) The ligature group exhibiting the suture thread (ST) closely to the sulcular epithelium (SE). It’s also observed the transition from oral epithelium to a hyperplastic sulcular epithelium and then, to junctional epithelium (JE). Note the associated intense chronic inflammation, mainly located in the subepithelial region (objective x10); D) Region of sulcular epithelium in high magnification being possible to observe the presence of microabscess (MA) and underlying, in the connective tissue, chronic inflammatory infiltrate composed mainly of lymphocytes (red arrows) (objective x20); E) Note the presence of suture thread ligature and the epithelium in close contact presenting lymphocyte exocytosis (objective x40); F) In this microscopic picture it is possible to observe the presence of the basophilic reverse lines **(black** arrows) in the alveolar bone near to the tooth. Note the absence of an evident osteoblast layer in the ligature group (objective x20).

To further characterize the pathology establishment, we performed microscopic analyses. The histopathology revealed that in the ligature group, an intense and disperse inflammatory infiltrate mainly composed of lymphocytes and neutrophils was observed in almost all cases. It is noteworthy that this inflammation extended until the epithelium (exocytosis) only in the ligature group (Fig. 5 and Table 1). Just one case from the control group presented a discrete and chronic inflammatory infiltrate composed of plasma cells and some lymphocytes located in the subepithelial area. Periodontal pocket was identified in most of the cases from the ligature group and it was especially noted near the suture thread used as the ligature tool (Fig. 5 and Table 1). All cases from the control group preserved the osteoblast layer whereas just one case from the ligature group maintain this layer (Fig. 5B and F and Table 1). However, it is important to emphasize that the loss of this layer does not necessarily indicate that osteoblasts are absent in this process, but there is an imbalance between bone resorption and apposition processes, with a predominance of bone resorption and, consequently, bone loss. It was also noticeable that in both groups there was basophilic reverse lines (Fig. 5F). Overall, the control group demonstrated discrete lymphoplasmacytic inflammatory infiltrate that is common including in the healthy gum due to dental trauma. The prevalence of this characterizes were quantified in Table 1.

**Table 1:** Quantitative analysis of groups comparing the microscopic periodontal hallmarks used in the study. The inflammation includes the presence of chronic inflammation, acute, moderate, intense, localized or diffuse.

TYPE OF INJURY	CONTROL GROUP %	LIGATURE GROUP %
PRESENCE OF INFLAMMATION	20	80
PRESENCE OF RESORPTION	0	100
PRESENCE OF OSTEOBLASTS	100	10
PERIODONTAL POCKET	0	70
PRESENCE OF EXOCYTOSIS	0	70

Together, these data demonstrate that the method here used to apply ligature in the first molar of mice was able to induce several hallmarks of periodontitis pathology, making it a suitable method to study this disease. The literature lacks cheap and easily accessible materials to reproduce the study in an experimental animal model for the analysis of periodontitis. In this case, the development of this work provides additional information that allows researchers greater access and reproducibility of this disease in relation to other studies in the literature.

## Funding

This work was supported by Conselho Nacional de Desenvolvimento Científico e Tecnológico and Fundação de Amparo à Pesquisa do Estado do Rio de Janeiro (FAPERJ- grant number E-26/010.002531/2016). This study was financed in part by the Coordenação de Aperfeiçoamento de Pessoal de Nível Superior - Brasil (CAPES) – Finance Code 001.
